# Sildenafil and suicide in Sweden

**DOI:** 10.1007/s10654-021-00738-4

**Published:** 2021-04-01

**Authors:** Ralph Catalano, Sidra Goldman-Mellor, Tim A. Bruckner, Terry Hartig

**Affiliations:** 1 School of Public Health, University of California, Berkeley, Berkeley, CA 94720 USA; 2 Program in Public Health, University of California, Merced, Merced, CA 95343 USA; 3 Program in Public Health, University of California, Irvine, Irvine, CA 92697 USA; 4 Institute for Housing and Urban Research, Uppsala University, 75120 Uppsala, Sweden

**Keywords:** Sildenafil, Sexual intimacy, Suicide

## Abstract

Much theory asserts that sexual intimacy sustains mental health. Experimental tests of such theory remain rare and have not provided compelling evidence because ethical, practical, and cultural constraints bias samples and results. An epidemiologic approach would, therefore, seem indicated given the rigor the discipline brings to quasi-experimental research. For reasons that remain unclear, however, epidemiologist have largely ignored such theory despite the plausibility of the processes implicated, which engender, for example, happiness, feelings of belonging and self-worth, and protection against depression. We use an intent-to-treat design, implemented via interrupted time-series methods, to test the hypothesis that the monthly incidence of suicide, a societally important distal measure of mental health in a population, decreased among Swedish men aged 50–59 after July 2013 when patent rights to sildenafil (i.e., Viagra) ceased, prices fell, and its use increased dramatically. The test uses 102 pre, and 18 post, price-drop months. 65 fewer suicides than expected occurred among men aged 50–59 over test months following the lowering of sildenafil prices. Our findings could not arise from shared trends or seasonality, biased samples, or reverse causation. Our results would appear by chance fewer than once in 10,000 experiments. Our findings align with theory indicating that sexual intimacy reinforces mental health. Using suicide as our distal measure of mental health further implies that public health programming intended to address the drivers of self-destructive behavior should reduce barriers to intimacy in the middle-aged populations.

## Introduction

Rewards beyond immediate physical gratification reinforce sexual intimacy among humans [1–4]. Intuition, theory and observational data suggest that these rewards include enhanced mental health, realized, for example, through greater happiness [1]; feelings of belonging, desirability, and self-worth [5, 6]; satisfaction with life [5, 7]; and protection against depression [5, 8]. Testing such hypotheses via experimentation has, however, proved difficult for several reasons including that ethical and practical constraints require recruitment of pre-existing couples as the unit of analysis. Couples differ on how much sexual intimacy they prefer [9]. Randomly assigning a representative sample of couples to low vs. high intimacy will, therefore, move many away from their optimal level, thereby lowering their subjective well-being as an artifact of the design. Indeed, the only published study based on randomization found that happiness *declined* among those assigned to more frequent coitus [9]. External validity of true experiments may also prove low because recruiting an unbiased sample of couples willing to comply with protocol will likely remain problematic.

Survey-based research [1, 4, 10, 11] would avoid many of the problems noted above if self-reporting of sexual behavior and well-being proved accurate and if answering questions about intimacy deterred fewer participants than does the prospect of complying with experimental protocols. Assuming those circumstances applied, a positive association between self-reported sexual activity and well-being would, however, provide only weak evidence of causation. The association could arise via reverse causation. For example, pre-existing depression could lead to reduced intimacy, and indeed the defining characteristics of depression include loss of libido [4].

Using self-reported appraisals of psychological states as outcomes also raises questions about the comparability and objectivity of survey-based research. Some research has used, for example, a measure of mood as an outcome [8, 9], while other studies have gauged life satisfaction [12] or quality of life [11], and still others measured domain-specific outcomes such as satisfaction with marriage or relationships [13]. Such measures may not, moreover, characterize anything of sufficient societal importance to warrant public health, as opposed to clinical, attention.

Epidemiologists, whose methods have often proved helpful when experimentation cannot produce compelling evidence, have not attempted to test the association between sexual intimacy and mental health. They have, however, asked whether sexual intimacy predicts mortality [14]. That literature has proved controversial for several reasons including that some scholars have made light of attempts to understand the association [15, 16].

We offer a test that avoids many of the problems noted above. We test the hypothesis that the monthly incidence of suicide, an unquestionably objective and societally important measure of mental health in a population [17, 18] decreased among Swedish men aged 50–59 after July 2013 when the patent rights to sildenafil (i.e., generic Viagra) ended. Swedish health insurance did not cover “lifestyle” drugs like Viagra, leaving consumers to pay the entire cost. After the patent rights terminated, major Swedish pharmacy chains sold 12 tablets of generic sildenafil (50 mg sildenafil) for as little as 300 SEK (about 45 USD at then-current exchange rates), about 25% of the cost for Viagra (1200 SEK, or about 180 USD) [16]. The number of Swedish men using sildenafil averaged 62,000 per year from 2007 to 2012. That number increased to 101,000 for the years 2014–2017 [20].

We focused our hypothesis on men aged 50–59 for two reasons. First, the prescribing of sildenafil to treat erectile dysfunction increased most quickly in that age group in Sweden [21] as in other countries [22, 23]. Second, data provided us by the Swedish National Board of Health and Welfare and described below show these men have historically accounted for more suicides in Sweden than have any other 10-year age by sex group.

We argue that if sexual intimacy boosts constituents of subjective well-being and protects against depression, the high incidence of suicide among Swedish men aged 50 to 59 occurs, at least in part, due to the onset of erectile dysfunction. If correct, this argument implies that the 63% increase in men using sildenafil likely reduced the historically expected fraction of vulnerable men in the population.

Biased samples do not affect our test because our data describe the entire population of Swedish men aged 50–59. No research protocols required individuals or couples to behave contrary to their preferences for intimacy. And reverse causation does not apply—suicide among middle-aged Swedes did not cause pharmaceutical firms to lower the price of sildenafil.

## Methods

### Data

We obtained sex specific monthly counts of suicide among Swedes from the registry of causes of death maintained by the Swedish National Board of Health and Welfare. Our test months began January 2005 and ended December 2014. A review of reliability assessments describes the Swedish suicide data as accurate [24].

We cannot compute a true monthly “rate” of suicide for Swedish men aged 50–59 because we do not know the monthly number at risk. We, instead, use the monthly counts of suicide. Epidemiologists typically prefer rates to counts because, among other reasons, an association between a population exposure and counts could arise when the population at risk decreases or increases coincident with exposure. The reduced number of suicides we hypothesize could not, however, follow from a decrease in men aged 50–59 living in Sweden when the price of sildenafil fell in 2013 and 2014. We assert this because annual estimates of men in this age group show *increases* in these two years [25].

### Analyses

Our study follows intent-to-treat logic based on instrumental variables [26, 27]. We consider the novel use of sildenafil by men aged 50–59 years as the “treatment” or X. Y is the monthly count of suicides. Z (“treatment assignment”) is the expiration of the Viagra patent in July 2013. Z serves as a valid estimator (“instrumental variable”) of the effect of X on Y because three conditions apply: Z precedes, and affects, X; Z affects Y only through X; and Z and Y share no common cause. Given these conditions, the Z → Y effect approximates the X → Y effect.

We implement intent-to-treat logic with an interrupted time-series test [28]. The test essentially asks whether the monthly counts of suicides among Swedish men aged 50–59 differed from statistically expected values after the introduction of low-priced sildenafil in July 2013. Tests of association typically specify the mean of all values as the value expected for any case. Variables measured over time, however, often exhibit “autocorrelation” in the form of secular trends, cycles, or the tendency to remain elevated or depressed, or to oscillate, after high or low values. The expected value for a case of an autocorrelated series is not its mean, but rather the value predicted by autocorrelation.

Following practice dating to Fisher [29], we solved this problem by identifying time-series models that best fit observed autocorrelation in suicides from January 2005 to June 2013 and by using the values predicted by these models for the next 18 months (i.e., those after the introduction of generic sildenafil) as counterfactuals. We used the most developed and widely disseminated type of such modeling. The method, devised by Box and Jenkins [30], identifies which of a very large family of models best fits serial measurements. The Box and Jenkins approach attributes autocorrelation to integration as well as to "autoregressive" and "moving average" parameters. Integration describes secular trends and strong seasonality. Autoregressive parameters best describe patterns that persist for relatively long periods, while moving average parameters parsimoniously describe less persistent patterns.

Our test proceeded through three steps. First, we used Box and Jenkins methods to detect and model autocorrelation in counts of suicide among Swedish men aged 50–59 for the 102 months preceding the introduction of low-priced sildenafil (i.e., January 2005–June 2013). Second, we applied the model with parameter values estimated in step 1 to the 120 months that included 18 post-introduction months (i.e., through December 2014). We reasoned that any effect of change in prices on prescribing should appear within 18 months given that 56% of Swedes report visiting primary care physicians in a year and another 30% report visits at slightly longer intervals [31]. The residuals (i.e., observed minus expected values) of this estimation measure the degree to which the observed values differed from those statistically expected. Third, we used the methods of Alwan and Roberts [32] to detect outlying sequences in the residuals of the model estimated in step 2. This method not only detects outlying values but also characterizes them as level shifts, single spikes, or spike-and-decay sequences. Our hypothesis predicts that the last 18 residuals will include a sequence of values detectably lower than expected.

## Results

**Fig. 1:**
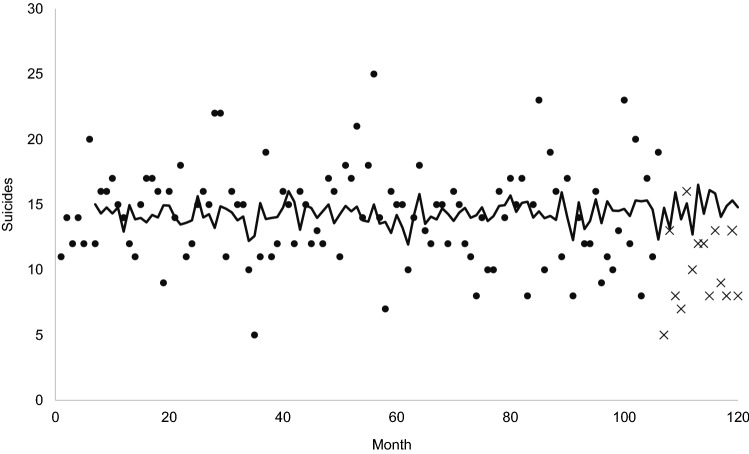
Observed (points) and expected (line) suicides among Swedish men aged 50–59 for 120 months beginning January 2005 and ending December 2014 (first 6 months of expected values lost to modeling). Lower than expected sequence of observed suicides marked with “X.”

Monthly suicides among Swedish men aged 50–59 ranged from 5 to 25 with a mean of 13.75 (standard deviation = 3.81) over our 120-month test period. The points in Fig. 1 show the 120 monthly counts of suicide.

Step 1, in which we identified autocorrelation in the series for the first 102 months (i.e., those before low-priced sildenafil) and specified the best fitting Box-Jenkins parameters, yielded the following model.

$${\text{Y}}_{{\text{t}}} = {14}.{26} + \left( {{1} - 0.{\text{23B}}^{{6}} } \right){\text{a}}_{{\text{t}}}$$

Y_t_ is the number of suicides among Swedish men age 50–59 in month t. The standard error for the constant, 14.26, was 0.26. The value 0.23 is a Box-Jenkins moving average parameter, the standard error for which is 0.10. B^6^ is the backshift operator or value of the error term (i.e., a at t − 6). The moving average parameter at 6 months indicates seasonality in the incidence of suicide among these Swedish men.

**Fig. 2:**
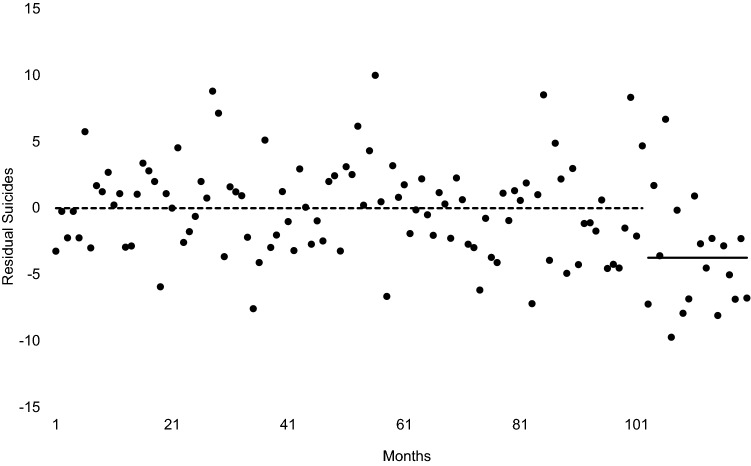
Observed less expected suicides among Swedish men aged 50–59 for 120 months beginning 1/2005 and ending 12/2014. Expected values derived from autocorrelation in 102 pre-generic sildenafil months. Pre-generic mean shown as dashed line, post-generic mean shown as solid line

Figures 1 and 2 show the results of step 2, in which we fit the model described above to all 120 months of the test period. The line in Fig. 1 traces the expected, or counterfactual, values while the points show the observed values. Figure 2 shows the model residuals or differences between the expected and observed values. The dotted line in Fig. 2 shows the mean of the residuals for the 102 pre-generic sildenafil months. The mean serves as the expected value of the 102-value series because the model estimated in step 1 removed autocorrelation. If the introduction of low-priced sildenafil had no effect on suicide, the mean of the 18 post-generic months would equal that of the prior 102 months. The solid line shows the mean of the residuals for the post-generic months. Consistent with the argument that suicides fell below expected values after the introduction of relatively inexpensive sildenafil, the differences between expected and observed values appear increasingly negative in the last 18 months.

Step 3, in which we applied outlier detection routines to the residuals of the model estimated in step 2, confirmed the results suggested by the figures. The post-generic downward shift in suicides appears statistically detectable beginning November 2013. The drop of approximately 4.6 suicides per month persists for the 14 months through the remainder of the test period (i.e., through December 2014). The model including the level shift was as follows.

$${\text{Y}}_{{\text{t}}} = {14}.{26} - {4}.{\text{66I}}_{{{\text{t}} - {5}}} + \left( {{1} - 0.{\text{23B}}^{{6}} } \right){\text{a}}_{{\text{t}}}$$

I is a binary variable scored 1 for the months November 2013–December 2014 and 0 otherwise. The decrease of − 4.66 had a standard error of 0.94. This estimate implies that about 65 fewer suicides than expected occurred among men aged 50–59 over the 14 months.

We repeated our test for men 40–49, men 60–69, all men other than those aged 50–59, and for women aged 50–59. We reasoned that any discovered inverse relation between the introduction of sildenafil and suicides would be attenuated (or null) in groups that do not use sildenafil, have lower baseline expectations of sexual intimacy involving erection, or lower suicide rates. None of these additional tests found negative outlying sequences in suicides in the 18 months following the introduction of low-cost sildenafil (see tabled results in “Appendix”). These findings indicate that the inverse relation appears specific to men aged 50–59 years.

The question of what happened after 2014 inevitably arises. Projecting beyond 2014 from parameters estimated for 2005–June 2013 strikes us as risky but we did so through 2016 (last year for which we could obtain suicide data at the time of our analyses) to estimate if, and when, the sildenafil association statistically dissipated. Although observed suicides remained consistently below expected from the 102 pre-generic sildenafil months, the outlier detection methods found that the difference became statistically undetectable, using *p* < 0.05 (1-tailed test) as the criterion for detection, in January 2015.

## Discussion

Our findings support the intuition and theory that sexual intimacy involving male erection protects against suicide among older men. Our methods ensure that this association could not arise from shared trends or seasonality, biased samples, or reverse causation. We further note that our finding would appear by chance fewer than once in 10 000 experiments (i.e., point estimate of − 4.66 with a standard error of 0.94) and that they align with theory indicating that sexual intimacy reinforces mental health.

Only replication can determine whether the association we found in Sweden describes other societies in which changes in patent rights affected the availability of sildenafil. We, however, know of no reason to suspect that the association would appear only in this cohort of Swedish men.

Although using suicide as an outcome connects our theory to an objective and important phenomenon, it likely leads to an underestimate of the association between low-priced sildenafil and mental health because suicide remains an extreme manifestation of diminished well-being. We could not, moreover, test for any unintended adverse health consequences (e.g., changes in sexually transmitted diseases).

Our methods do not estimate the efficacy of sildenafil in reducing an individual’s suicide risk. We assume that the rapid increase in the use of sildenafil among Swedish men in late 2013 arose in large part due to the availability of low-priced generic sildenafil. We, however, could not access monthly information on price, volume of prescriptions, or age of patients receiving these prescriptions. An important extension of our study would involve such data as well as longitudinal information on men’s mental health and likely mediating processes before and after July 2013. Such data would allow individual-level tests of whether and how the novel use of sildenafil improved mental health among men in their 50′s.

We speculate that the difference between expected and observed suicides declined below statistically detectable levels, although did not disappear, in 2015 for at least two reasons. The first assumes that lowering the price of sildenafil in 2013 likely reduced erectile dysfunction among men regardless of age. When men younger than 50 in 2013 eventually aged past 50, the sequelae of erectile dysfunction, including an increased risk of suicide, appeared lower among them than among men 50 to 59 when the price of sildenafil dropped.

Second, counterfactuals in tests that, like ours, use autocorrelation to arrive at expected values eventually “adjust” for interruptions with persistent effects. The effects, in other words, become statistically expected. Our test, for example, found autocorrelation such that an unexpected value at month t influences expectations for month t + 6. Our counterfactuals for 2015, therefore, reflected not only the pre-generic price months but also the lower-than-expected values observed in 2013 and 2014. We note, however, that the alternative approach of using forecasts in 2013 and 2014 to estimate expected values farther in the future would provide no more certainty than our approach because detection intervals in such models expand relatively quickly.

We did not hypothesize a detectable decline in suicide among women aged 50 to 59 because underlying causal processes, such as the etiology of depression [33], may differ between women and men. The failure to find an association, however, requires comment if for no other reason than sexual intimacy also relates to the mental health of women [7, 8]. Although the comparatively low incidence of suicide among women makes detecting differences over time less certain, we note that the null finding could arise from differences between men and women in the purpose and meaning of male erection as a component of intimacy at this stage of life [7]. For example, having and maintaining an erection may contribute directly to a man’s confidence and self-esteem [5], while insofar as this contributes to such self-perceptions among a female partner it would do so indirectly, as through a sense of desirability [34], with the respective effects possibly changing differentially with age across the genders [35]. The present data, however, do not enable us to do more than speculate on such possibilities. We can only acknowledge the complexity of the individual and contextual determinants of suicide rates and the need for further attention to the role that sexual intimacy plays in suicide, as encouraged by our strong epidemiologic findings.

Our results appear consistent with the general argument that many people enjoy sexual intimacy as a fundamental component of relationships that confer wellbeing and good mental health. Means to overcome erectile dysfunction could, therefore, promote restoration of a relational resource diminished by an inability to share desired forms of intimacy. These means likely include not only pharmaceutical treatment, but also individual or couple’s counselling, behavioral therapy [36], or even public policies (e.g., vacation legislation) that allow people more time for sexual intimacy. Insofar as salutary relationships remain important not only for the partners but also for the people around them and for the organizations to which they contribute, interventions such as greater access to sildenafil likely produce benefits to society.

## Data Availability

Publicly available.

## Appendix

See Tables 1, 2, 3, 4.

**Table 1:** Estimated parameters for time-series equation predicting monthly counts of suicides among Swedish men 40 to 49 years old, for 102 months spanning January 2005 to December 2014 (standard errors in parentheses)

Parameter	Point estimate	SE
Constant	11.54	(0.33)
Autoregression	None	–
Moving average	None	–

Outlier identification and estimation routines detected one negative outlier—a spike and decay sequence starting November 2007: coef. = − 6.60; SE = 2.23, *p* < 0.01

**Table 2:** Estimated parameters for time-series equation predicting monthly counts of suicides among Swedish men 60–69 years old, for 102 months spanning January 2005–December 2014 (standard errors in parentheses)

Parameter	Point estimate	SE
Constant	10.97	(0.50)
Autoregressive	None	–
Moving average at t + 12	− 0.35	(0.10)

Outlier identification and estimation routines detected two positive spike and decay outlying sequences. One in April 2010: coef. = 11.43; SE = 3.77, *p* < 0.01, and another in June 2010: coef. = 11.86; SE = 3.78, *p* < .01

**Table 3:** Estimated parameters for time-series equation predicting monthly counts of suicides among adult Swedish men other than 50–59 years old, for 102 months spanning January 2005 to December 2014 (standard errors in parentheses)

Parameter	Point estimate	SE
Constant	52.43	(0.90)
Autoregression	None	–
Moving average	None	–

Outlier identification and estimation routines detected no outliers

**Table 4:** Estimated parameters for time-series equation predicting monthly counts of suicides among Swedish women 50–59 years old, for 102 months spanning January 2005 to December 2014 (standard errors in parentheses)

Parameter	Point estimate	SE
Constant	5.35	(0.20)
Autoregression	None	–
Moving average	None	–

Outlier identification and estimation routines detected one positive spike outlier in August 2014: coef. = 8.67; SE = 2.08, *p* < 0.0001

## Code availability

From corresponding author upon request.
